# Diagnostic value of multi-regional multi-parameter transcranial sonography in differentiating Parkinson’s disease from essential tremor

**DOI:** 10.3389/fneur.2026.1860398

**Published:** 2026-08-14

**Authors:** Hong Yao, Weiwei Wang, Canfang Hu, Xuelin Liang, Lei Zhang

**Affiliations:** 1 Department of Ultrasound Medicine, Jinshan Central Hospital Affiliated to Shanghai University of Medicine & Health Sciences, Shanghai, China; 2 Department of Public Health, Jinshan Central Hospital Affiliated to Shanghai University of Medicine & Health Sciences, Shanghai, China; 3 Department of Neurology, Jinshan Central Hospital Affiliated to Shanghai University of Medicine & Health Sciences, Shanghai, China

**Keywords:** combined predictor, differential diagnosis, essential tremor, Parkinson’s disease, substantia nigra, transcranial sonography

## Abstract

**Objective:**

Differentiating Parkinson’s disease (PD) from essential tremor (ET) remains challenging during early clinical stages. This study aimed to evaluate the diagnostic value of multi-regional multi-parameter transcranial sonography (TCS) in differentiating PD from ET.

**Methods:**

A total of 80 PD patients and 23 ET patients admitted to the Department of Neurology between January 2024 and December 2025 were enrolled. All participants underwent comprehensive clinical assessment and standardized TCS examination. TCS parameters included bilateral substantia nigra (SN) hyperechogenicity area, bilateral midbrain area, third ventricle width, lentiform nucleus echogenicity, and brainstem raphe echogenicity. Age-adjusted binary logistic regression with bootstrap internal validation, Firth penalized-likelihood sensitivity analysis, and repeated cross-validation was performed.

**Results:**

The bilateral SN hyperechogenicity total area (SNsum) was significantly larger in the PD group [median 0.30 (0.13–0.46) cm^2^] compared with the ET group [median 0 (0–0.25) cm^2^, *p* < 0.001]. Third ventricle width was significantly greater in PD patients (7.31 ± 1.73 mm vs. 5.57 ± 1.47 mm, *p* < 0.001). Age-adjusted logistic regression identified SNsum (OR = 69.023, *p* = 0.014) and third ventricle width (OR = 2.531, *p* = 0.001) as independent predictors. The combined predictor achieved an area under the ROC curve (AUC) of 0.848 (95% CI: 0.743–0.932), which was significantly higher than SNsum alone (AUC = 0.739; DeLong *p* = 0.022) and third ventricle width alone (AUC = 0.783; DeLong *p* = 0.034). At the optimal cutoff the combined predictor yielded a sensitivity of 75.0% (95% CI: 64.5–83.2%) and specificity of 82.6% (95% CI: 62.8–93.0%). Bootstrap-corrected AUC was 0.830, and the Firth penalized model and repeated cross-validation (AUC = 0.812) yielded concordant estimates.

**Conclusion:**

Multi-regional multi-parameter TCS may serve as a practical neuroimaging tool for differentiating PD from ET. The combined predictor provided moderate, internally validated discrimination and should be regarded as an exploratory tool requiring confirmation in larger, multicenter, prospective cohorts with external validation before clinical application.

## 1 Introduction

Parkinson’s disease (PD) is the second most prevalent neurodegenerative disorder worldwide, characterized primarily by motor symptoms including bradykinesia, resting tremor, and rigidity (1). With advancing diagnostic capabilities and the acceleration of global population aging, the number of individuals affected by PD has steadily increased, representing a significant public health burden (1). Essential tremor (ET) is among the most common movement disorders in middle-aged and elderly populations, clinically defined by postural and kinetic tremor predominantly affecting the upper extremities, occasionally accompanied by head, orofacial, or voice tremor (2). The early clinical manifestations of PD and ET frequently overlap, and a subset of ET patients present with additional neurological features (termed ET-plus), which has been increasingly recognized as a potential risk factor for subsequent progression to PD (3). This phenotypic convergence poses considerable challenges for accurate differential diagnosis, particularly in the early disease stages. Clinico-pathological studies have demonstrated that diagnostic error rates can reach up to 25% in community-based settings when relying solely on clinical criteria (4), underscoring the urgent need for objective biomarkers and complementary imaging tools.

The differential diagnosis between PD and ET currently lacks disease-specific biomarkers. Clinical diagnosis relies predominantly on established diagnostic criteria based on symptom profiles and disease history (5, 6). However, interpretation bias regarding postural versus resting tremor and slowly progressive motor dysfunction can lead to misdiagnosis, directly affecting treatment strategies and prognostic assessments (4). Advanced neuroimaging modalities, including dopamine transporter single photon emission computed tomography (DaT-SPECT) and positron emission tomography (PET), have demonstrated high diagnostic specificity for PD (7). Nevertheless, these techniques are constrained by prolonged acquisition times, radiotracer exposure, high costs, and limited availability in primary care settings, substantially restricting their clinical applicability for routine screening and differential diagnosis (7, 8). Conventional cranial computed tomography and magnetic resonance imaging (MRI) are more readily accessible but demonstrate limited specificity for differentiating PD from ET in the absence of structural abnormalities (9). Therefore, there exists a critical unmet need for cost-effective, non-invasive, and widely accessible neuroimaging tools that can complement clinical evaluation in the differential diagnosis of tremor syndromes.

Transcranial sonography (TCS) has emerged as a promising neuroimaging modality for movement disorder evaluation, offering advantages of real-time imaging, non-invasiveness, absence of ionizing radiation, and favorable cost-effectiveness (10). Since Becker and colleagues first described the characteristic hyperechogenicity of the substantia nigra (SN) in PD patients using TCS in 1995 (11), extensive research has confirmed SN hyperechogenicity as a robust biomarker for PD (12). A recent systematic review and meta-analysis demonstrated that SN-TCS achieves pooled sensitivity and specificity values exceeding 80% for discriminating PD from healthy controls (12). However, the diagnostic accuracy of SN-TCS in differentiating PD specifically from ET has shown considerable variability across studies, influenced by sample sizes, ethnic differences, equipment specifications, and operator expertise (13). With continuous technological advances, contemporary TCS platforms equipped with standardized parameters and high-end transducers can now achieve high-resolution imaging of deep brain structures, enabling systematic evaluation of not only the midbrain SN but also the brainstem raphe (BR) at the midbrain level, the lentiform nucleus (LN) at the thalamic level, third ventricle (TV) width, and temporal horn indices (14–16).

Despite the expanding repertoire of TCS-detectable parameters, relatively few studies have systematically incorporated multiple brain regions and parameters for the differential diagnosis of PD and ET, and existing findings remain somewhat controversial (17). Most prior investigations have focused exclusively on SN echogenicity as the sole diagnostic criterion, potentially limiting overall diagnostic sensitivity and overlooking the complementary information provided by other TCS-accessible structures. Recent work integrating multimodal neuroimaging and artificial-intelligence approaches—such as multimodal brain-network topology analysis (18) and self-supervised vision foundation models applied to routine multi-contrast MRI (19)—has shown that combining complementary imaging features markedly improves the computer-aided diagnosis of parkinsonian disorders, providing a conceptual rationale for adopting a multi-parameter framework within a single, widely accessible modality such as TCS. Furthermore, the majority of existing studies have not employed rigorous statistical methods for constructing combined predictive models, such as age-adjusted multivariable regression with appropriate internal validation. The present study aimed to evaluate a comprehensive multi-regional, multi-parameter TCS protocol encompassing SN, BR, LN, and TV measurements, and to construct a combined predictor model integrating independently significant TCS parameters using age-adjusted binary logistic regression. The specific novelty of this work lies in the simultaneous acquisition of multiple regional TCS parameters within one examination and their formal integration into an age-adjusted combined predictor whose incremental value over single parameters was quantified by pairwise DeLong testing and whose stability was examined by bootstrap resampling, Firth penalized-likelihood regression, and repeated cross-validation. The model’s discriminative performance was assessed through receiver operating characteristic (ROC) curve analysis with pairwise DeLong tests for statistical comparison of AUC values, and internal validity was evaluated using bootstrap resampling. This approach seeks to enhance the diagnostic performance of TCS in differentiating PD from ET and provide evidence-based guidance for early clinical identification.

## 2 Materials and methods

### 2.1 Study population

This retrospective study enrolled 120 patients with PD or ET who were admitted to the Department of Neurology (inpatient and outpatient) at Shanghai Sixth People’s Hospital Jinshan Branch between January 2024 and December 2025. Inclusion criteria were as follows: (1) age greater than 18 years; (2) ability to cooperate with TCS examination and completion of clinical questionnaires; (3) PD diagnosis meeting the MDS Clinical Diagnostic Criteria for Parkinson’s Disease (5); and (4) ET diagnosis conforming to the Consensus Statement on the Classification of Tremors from the International Parkinson and Movement Disorder Society (6). Exclusion criteria included: (1) severe psychiatric illness or cognitive impairment precluding assessment compliance; (2) prior cranial surgery including deep brain stimulation or ablative procedures; (3) severe comorbidities involving cerebrovascular, hepatic, renal, or pulmonary organs; and (4) inadequate temporal bone acoustic window on one or both sides during TCS examination.

All patients underwent comprehensive neurological examination, and diagnoses were jointly confirmed by two senior attending physicians with subspecialty expertise in movement disorders. Among the 120 initially screened patients, 10 (8.3%) were excluded due to poor temporal bone acoustic windows precluding adequate TCS imaging, and 7 (5.8%) were excluded for inability to complete clinical assessments. The final study cohort comprised 103 patients: 80 patients with PD (PD group) and 23 patients with ET (ET group). As this was an exploratory, single-center diagnostic study, no formal *a priori* sample size calculation was performed; all consecutive eligible patients within the enrollment period were included, and the resulting sample and group imbalance are addressed explicitly as limitations. This study was approved by the Ethics Committee of Jinshan Central Hospital affiliated to Shanghai University of Medicine & Health Sciences (Ethics number: jszxyy202413). Written informed consent was obtained from all participants.

### 2.2 Clinical data collection

Standardized data collection forms were used to record baseline demographics and clinical information for all participants, including age, sex, medical and family history, age at disease onset, and disease duration. For PD patients, the Unified Parkinson’s Disease Rating Scale Part III (UPDRS-III) motor score and Hoehn-Yahr (H-Y) stage were additionally assessed. All subjects underwent the complete multi-regional, multi-parameter TCS examination protocol.

### 2.3 TCS examination and parameter acquisition

TCS examinations were performed using a Philips EPIQ7 ultrasound system equipped with a 1–3 MHz sector transducer. The dynamic range was set at 45–55 dB, and the imaging depth was 14–16 cm. Time-gain compensation and image brightness were adjusted individually to achieve optimal two-dimensional image quality. All examinations were conducted by sonographers with more than five years of TCS experience who underwent standardized training prior to study initiation and were blinded to the clinical diagnoses and motor assessments of the participants. Final reports were confirmed jointly by the ultrasound and neurology departments. The TCS protocol followed the standardized procedure established at the 9th Meeting of the European Society of Neurosonology and Cerebral Hemodynamics (10).

Patients were positioned in left lateral decubitus, and the transducer was placed parallel to the ipsilateral orbitomeatal line against the temporal bone window to scan the midbrain; the contralateral side was examined identically. Imaging parameters were assessed at the following anatomical levels: midbrain, substantia nigra, thalamic-level lentiform nucleus, brainstem raphe, and third ventricle.

#### 2.3.1 Midbrain and substantia nigra assessment

**Figure 1:**
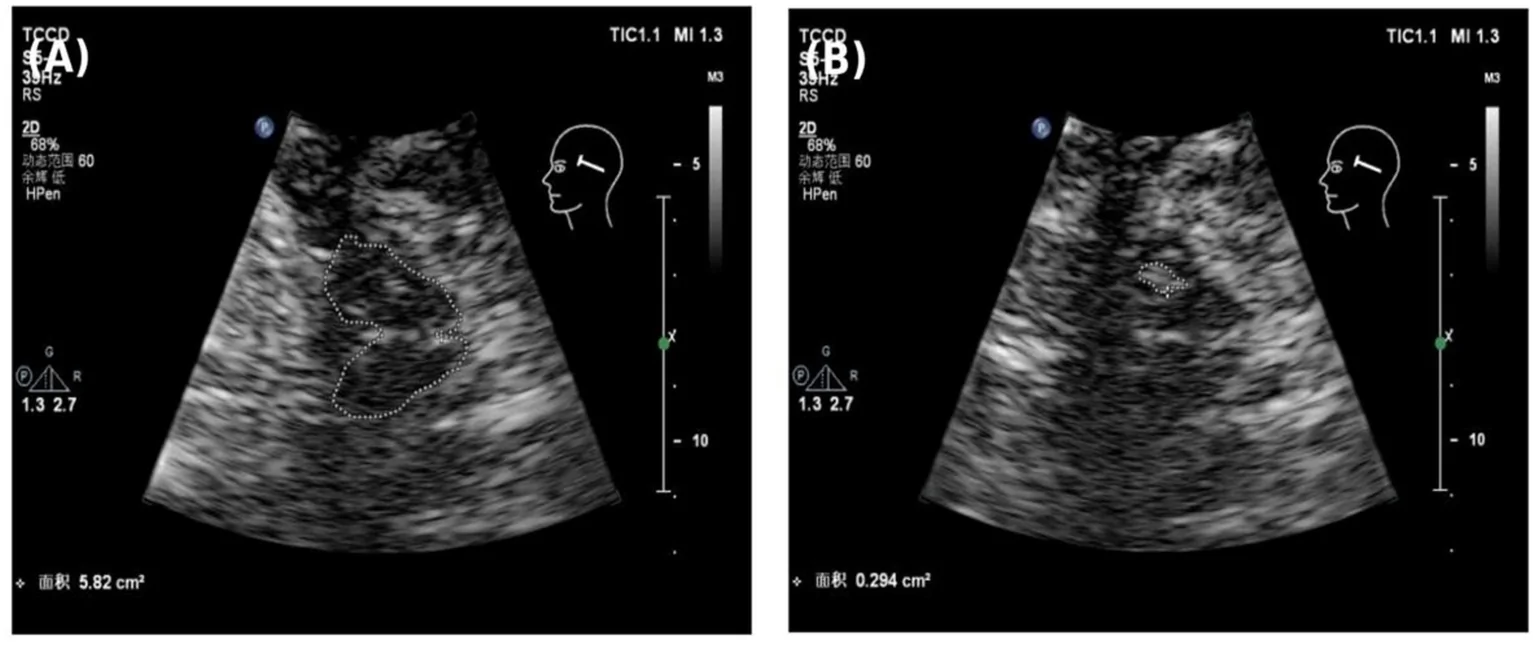
Transcranial sonography of the midbrain and substantia nigra in a PD patient. **(A)** Midbrain area measurement (total area = 5.82 cm^2^); **(B)** substantia nigra hyperechogenicity area measurement (area = 0.294 cm^2^).

The midbrain appeared as a butterfly-shaped hypoechoic structure surrounded by the hyperechoic interpeduncular cistern, with the SN located lateral to the red nucleus. When the maximal SN area was identified, the image was frozen and magnified. SN echogenicity was graded on a 5-point scale: Grade 1, homogeneous low echogenicity; Grade 2, scattered punctate or fine linear slightly enhanced echogenicity; Grade 3, patchy hyperechogenicity below the level of interpeduncular cistern echogenicity; Grade 4, patchy hyperechogenicity approximately equal to cistern echogenicity; Grade 5, patchy hyperechogenicity exceeding cistern echogenicity. For grades 3 and above, the hyperechoic area was manually traced along the ipsilateral SN border. The left and right SN hyperechogenicity areas (SN-L and SN-R, cm^2^) were recorded separately. Derived parameters included the maximum SN hyperechogenicity area (max[SN-L, SN-R]), total SN hyperechogenicity area (SNsum = SN-L + SN-R), mean midbrain area (Msum) calculated from bilateral measurements, and the SN-to-midbrain ratio (S/M ratio = SNsum/Msum, expressed as a percentage). Representative TCS images of midbrain and SN assessment are shown in Figure 1.

#### 2.3.2 Assessment of other regions and parameters

**Figure 2:**
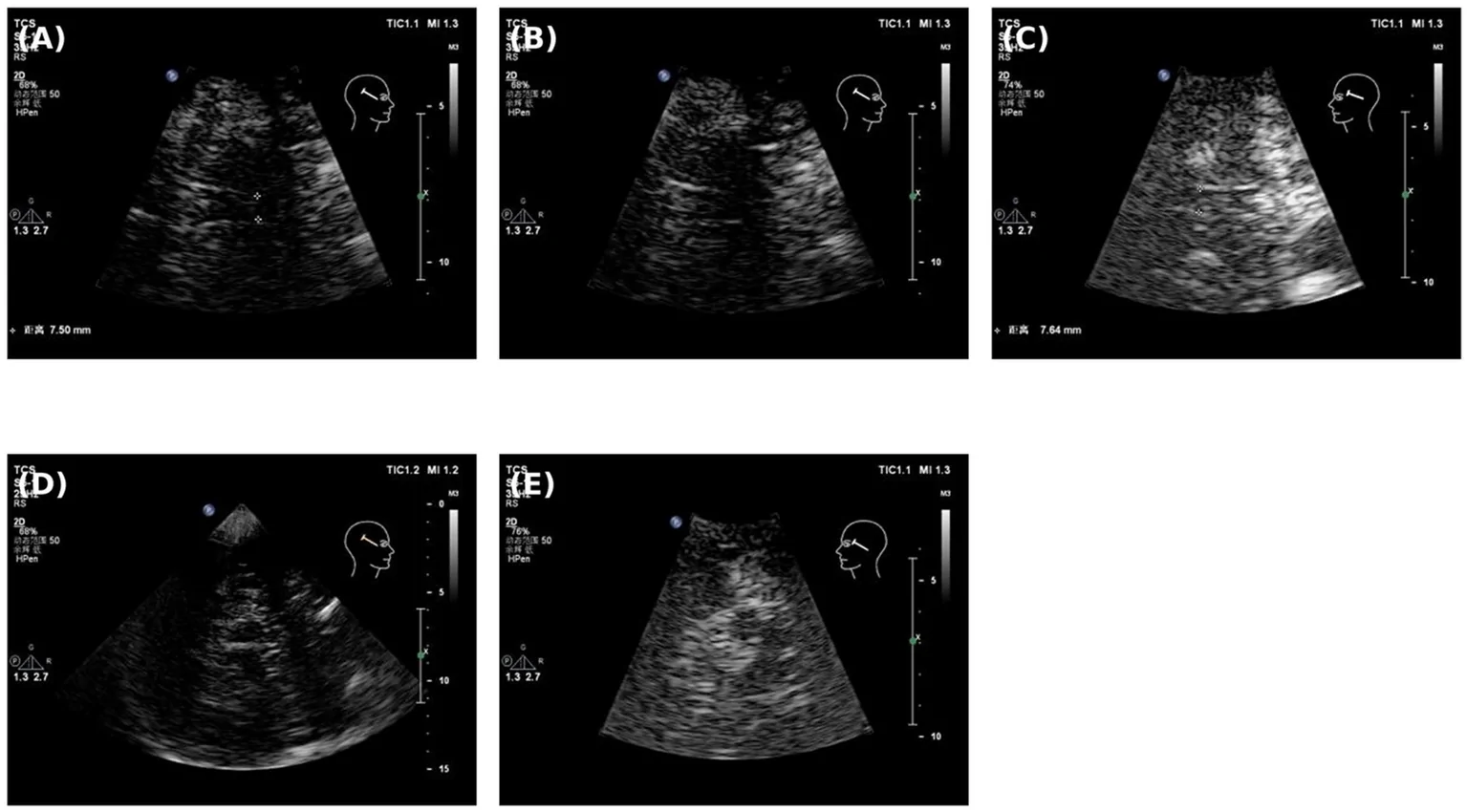
Transcranial sonography of the third ventricle, lentiform nucleus, and brainstem raphe. **(A)** Third ventricle width measurement. **(B)** Lentiform nucleus with homogeneous low echogenicity (negative). **(C)** Lentiform nucleus with patchy hyperechogenicity (positive). **(D)** Normal brainstem raphe echogenicity (negative). **(E)** Interrupted brainstem raphe echogenicity (grade 0).

By tilting the transducer approximately 10 degrees cephalad from the midbrain plane, the ventricular level and pineal gland were visualized. The third ventricle appeared as a structure between two parallel hyperechoic lines cephalad to the pineal gland, and its width was measured as the minimal distance between the inner edges of these two lines (in mm). Normal third ventricle width was defined as less than 7.0 mm based on established normative (16). The thalami were identified as hypoechoic structures on both sides of the third ventricle. The lentiform nucleus was visualized as a hypoechoic structure anterolateral to the thalamus, narrower medially and wider laterally. Lentiform nucleus hyperechogenicity was defined as present when the echogenicity of the LN region was higher than the surrounding brain parenchyma. At the midbrain plane, the brainstem raphe appeared as a continuous hyperechoic line at the center of the image. Raphe echogenicity was classified dichotomously: Grade 0, reduced, interrupted, or absent echogenicity; Grade 1, normal echogenicity equivalent to the red nucleus. Representative TCS images of these parameters are shown in Figure 2.

### 2.4 Statistical analysis

Statistical analyses were performed using IBM SPSS version 26.0 and R version 4.3.1. Continuous variables were assessed for normality using the Shapiro–Wilk test. Variables conforming to normal distribution were compared between groups using independent-samples t-tests (with Levene’s test for equality of variances) and expressed as mean ± standard deviation (SD). Non-normally distributed data were compared using the Mann–Whitney U test and presented as median (interquartile range, IQR). Categorical variables were expressed as frequencies and percentages, and between-group comparisons were performed using the chi-square test or Fisher’s exact test when the chi-square test conditions were not satisfied. Given the nine simultaneous comparisons of TCS parameters, Bonferroni correction was applied, yielding an adjusted significance threshold of *p* < 0.006 (0.05/9).

To identify independent TCS predictors of PD diagnosis, binary logistic regression analysis was performed. Prior to model construction, multicollinearity among SN-related variables was assessed using variance inflation factor (VIF) analysis. VIF values exceeding 10 indicated substantial collinearity, and only representative variables with acceptable VIF values were entered into the multivariable model. Given that third ventricle width increases physiologically with age and that age differed marginally between groups (*p* = 0.082), age was included as a covariate in the multivariable logistic regression to adjust for potential confounding. Model calibration was assessed using the Hosmer-Lemeshow goodness-of-fit test. Receiver operating characteristic (ROC) curve analysis was conducted to evaluate the diagnostic performance of individual and combined predictors. The combined predictor was derived from the logistic regression model using the predicted probability of PD diagnosis. Pairwise comparisons between AUC values were performed using the DeLong test (20). At the optimal cutoff defined by the maximum Youden index, the confusion matrix was tabulated and sensitivity, specificity, positive and negative predictive values, and accuracy were reported with 95% Wilson score confidence intervals. Internal validation of the combined predictor model was performed using bootstrap resampling (1,000 iterations) to estimate optimism and derive the optimism-corrected AUC (21). To address the low events-per-variable ratio and the wide confidence intervals arising from the small ET group, two prespecified sensitivity analyses were conducted: Firth penalized-likelihood logistic regression to obtain bias-reduced coefficient estimates under sparse-data conditions, and repeated stratified 10-fold cross-validation (five repeats) to estimate cross-validated discrimination. An additional age-stratified analysis restricted to participants aged 65 years or older was performed to examine residual age-related confounding of third ventricle width. A two-sided *p* value of 0.05 or less was considered statistically significant for hypothesis testing, unless otherwise specified for multiple comparison adjustments.

## 3 Results

### 3.1 Demographic and clinical characteristics

The study enrolled 80 PD patients and 23 ET patients. The PD group had a mean age of 71.31 ± 7.46 years (range 52–86 years) with 36 females (45.0%) and 44 males (55.0%), a median disease duration of 39 (12–72) months, H-Y stage greater than 2 in 48 patients (60.0%), and a median UPDRS-III score of 25 (14–42). The ET group had a mean age of 68.09 ± 8.80 years (range 49–84 years) with 15 females (65.2%) and 8 males (34.8%), and a median disease duration of 37 (10–71) months. No statistically significant differences were observed between the two groups regarding age (t = −1.754, *p* = 0.082), sex distribution (χ^2^ = 2.921, *p* = 0.087), or disease duration (Z = −1.258, *p* = 0.187). The demographic and clinical characteristics are summarized in Table 1.

**Table 1:** Demographic and clinical characteristics of the study population.

Variable	ET group (*n* = 23)	PD Group (*n* = 80)	t/Z/χ^2^	*p*
Age (years, mean ± SD)	68.09 ± 8.80	71.31 ± 7.46	−1.754	0.082
Age range (years)	49–84	52–86	—	—
Female, *n* (%)	15 (65.2)	36 (45.0)	2.921	0.087
Duration (months, M, IQR)	37 (10–71)	39 (12–72)	−1.258	0.187
H-Y stage > 2, *n* (%)	—	48 (60.0)	—	—
UPDRS-III (M, IQR)	—	25 (14–42)	—	—

ET, essential tremor; PD, Parkinson’s disease; H-Y, Hoehn-Yahr; UPDRS-III, Unified Parkinson’s Disease Rating Scale Part III; M, median; IQR, interquartile range; SD, standard deviation. “—” indicates not applicable.

### 3.2 Comparison of TCS parameters between groups

Shapiro–Wilk tests revealed that SN-related variables (SN-R, SN-L, SNsum, max[SN-L, SN-R], S/M ratio) were non-normally distributed in both groups (all *p* < 0.001), while mean midbrain area (Msum) and third ventricle (TV) width followed normal distributions in both groups (all *p* > 0.05). Levene’s tests confirmed homogeneity of variance for Msum (*F* = 2.145, *p* = 0.146) and TV width (*F* = 0.441, *p* = 0.508), supporting the use of independent-samples t-tests for these variables.

After applying Bonferroni correction (adjusted *α* = 0.006), significant differences were observed between the PD and ET groups across multiple TCS parameters. Regarding SN ultrasonography, the PD group demonstrated significantly higher right SN hyperechogenicity area (SN-R), maximum hyperechogenicity area, total hyperechogenicity area (SNsum), and SN-to-midbrain ratio compared with the ET group (all *p* < 0.006). Left SN hyperechogenicity area (SN-L) showed a trend toward significance (*p* = 0.006). For other TCS parameters, PD patients exhibited significantly greater third ventricle width (*p* < 0.001). Lentiform nucleus hyperechogenicity was more prevalent in the PD group (33.8% vs. 8.7%; Fisher’s exact test *p* = 0.019), though this did not survive Bonferroni correction. No statistically significant differences were found between groups in mean midbrain area (*p* = 0.620) or brainstem raphe echogenicity Grade 0 rate (Fisher’s exact test *p* = 0.543). The detailed comparison of TCS parameters is presented in Table 2.

**Table 2:** Comparison of TCS parameters between the PD and ET groups.

Variable	ET group (*n* = 23)	PD group (*n* = 80)	t/Z	*p*
SN-R (cm^2^; M, IQR)	0 (0–0)	0.14 (0–0.22)	−3.699	< 0.001*
SN-L (cm^2^; M, IQR)	0 (0–0.12)	0.16 (0–0.27)	−2.773	0.006
max[SN-L, SN-R] (cm^2^; M, IQR)	0 (0–0.16)	0.20 (0.11–0.30)	−3.404	0.001*
SNsum (cm^2^; M, IQR)	0 (0–0.25)	0.30 (0.13–0.46)	−3.520	< 0.001*
Msum (cm^2^; mean ± SD)	5.76 ± 0.67	5.66 ± 0.90	0.498	0.620
S/M ratio (%; M, IQR)	0 (0–4.35)	4.98 (2.33–7.62)	−3.512	< 0.001*
TV width (mm; mean ± SD)	5.57 ± 1.47	7.31 ± 1.73	−4.383	< 0.001*
LN hyperecho., *n* (%)	2 (8.7)	27 (33.8)	—	0.019†
BR Grade 0, *n* (%)	5 (21.7)	13 (16.3)	—	0.543†

*Significant after Bonferroni correction (adjusted α = 0.006). †Fisher’s exact test. SN, substantia nigra; SN-R, right SN hyperechogenicity area; SN-L, left SN hyperechogenicity area; SNsum, total SN hyperechogenicity area; Msum, mean midbrain area; S/M ratio, SN-to-midbrain ratio; TV, third ventricle; LN, lentiform nucleus; BR, brainstem raphe; M, median; IQR, interquartile range; SD, standard deviation.

### 3.3 Multicollinearity assessment and binary logistic regression

VIF analysis of SN-related parameters demonstrated substantial multicollinearity: SN-R (VIF = 14.000), SN-L (VIF = 15.056), SNsum (VIF = 18.341), max[SN-L, SN-R] (VIF = 12.657), and S/M ratio (VIF = 27.397). Given these findings, SNsum was selected as the representative SN variable for multivariable modeling, as it integrates bilateral information and demonstrated the strongest univariate association with PD diagnosis.

Multivariable binary logistic regression was performed with PD diagnosis as the dependent variable (ET = 0, PD = 1), including SNsum, third ventricle width, lentiform nucleus hyperechogenicity (coded as present/absent), and age as covariates. The results demonstrated that higher SNsum (OR = 69.023, 95% CI: 2.339–2037.215, *p* = 0.014) and greater third ventricle width (OR = 2.531, 95% CI: 1.472–4.354, *p* = 0.001) were independently associated with PD diagnosis after age adjustment, whereas lentiform nucleus hyperechogenicity (OR = 3.471, 95% CI: 0.566–21.277, *p* = 0.179) and age (OR = 0.946, 95% CI: 0.873–1.025, *p* = 0.172) did not achieve statistical significance. The Hosmer-Lemeshow goodness-of-fit test indicated adequate model calibration (χ^2^ = 11.046, *p* = 0.199). The events-per-variable ratio was 4.6 (23 ET events for 5 candidate variables). The results are presented in Table 3.

**Table 3:** Age-adjusted binary logistic regression analysis of TCS parameters predicting PD diagnosis.

Variable	β	SE	Wald	*p*	OR	95% CI	VIF
SNsum	4.234	1.727	6.012	0.014*	69.023	2.339–2037.215	1.175
TV width	0.929	0.277	11.265	0.001*	2.531	1.472–4.354	1.255
LN hyperecho.	1.244	0.925	1.810	0.179	3.471	0.566–21.277	1.111
Age	−0.056	0.041	1.862	0.172	0.946	0.873–1.025	1.311
Constant	−1.764	2.361	0.559	0.455	0.171	—	—

*p < 0.05. β, regression coefficient; SE, standard error; OR, odds ratio; CI, confidence interval; VIF, variance inflation factor; SNsum, total substantia nigra hyperechogenicity area; TV, third ventricle; LN, lentiform nucleus.

### 3.4 ROC curve analysis and internal validation

**Figure 3:**
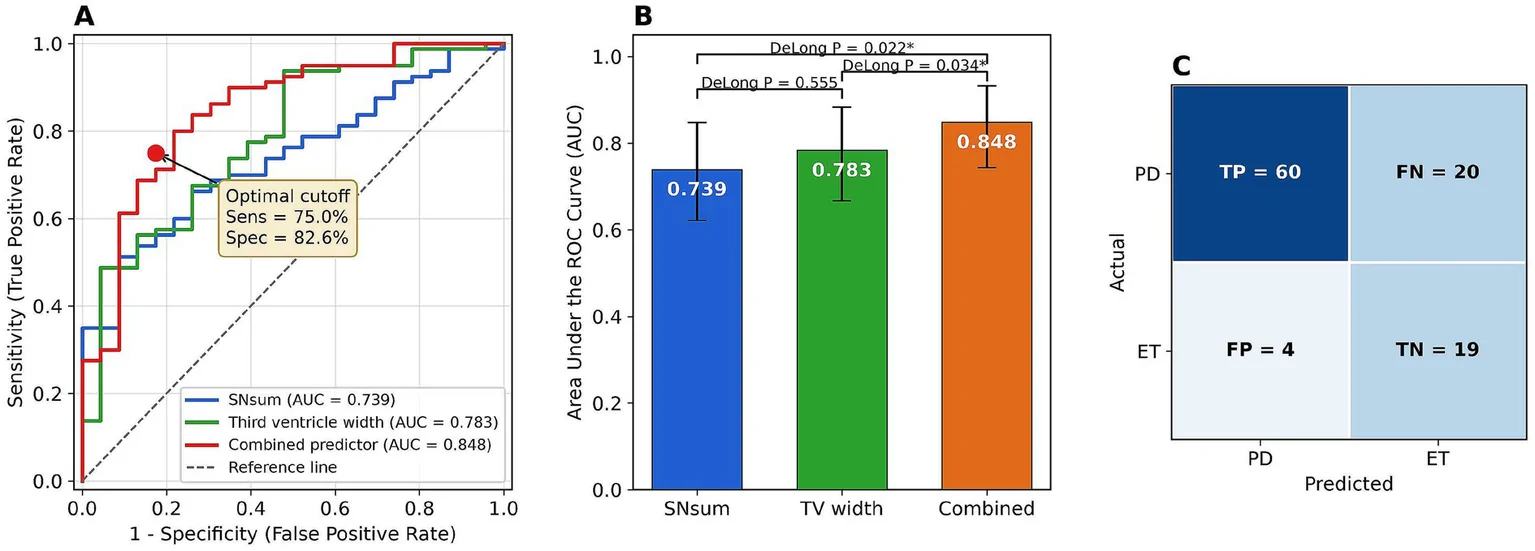
Receiver operating characteristic (ROC) curves of individual and combined TCS predictors for PD diagnosis. **(A)** Empirical (non-smoothed) step-like ROC curves are plotted for SNsum (AUC = 0.739), third ventricle width (AUC = 0.783), and the combined predictor (AUC = 0.848). The optimal cutoff point on the combined predictor curve is indicated, corresponding to a sensitivity of 75.0% and specificity of 82.6%. **(B)** Bar chart comparing AUC values with 95% confidence intervals and pairwise DeLong test results. DeLong test confirmed significantly higher AUC for the combined predictor compared with each individual parameter. **(C)** Confusion matrix of the combined predictor at the Youden-optimal cutoff (60 true positives, 20 false negatives, 4 false positives, 19 true negatives).

ROC curve analysis was performed to evaluate the diagnostic performance of independently significant predictors (Figure 3). The AUC for SNsum in predicting PD was 0.739 (95% CI: 0.622–0.847, *p* < 0.001), and the AUC for third ventricle width was 0.783 (95% CI: 0.667–0.883, *p* < 0.001). The combined TCS predictor, derived from the predicted probability of the age-adjusted logistic regression model, achieved an AUC of 0.848 (95% CI: 0.743–0.932, *p* < 0.001). Pairwise DeLong tests confirmed that the combined predictor achieved a significantly higher AUC than SNsum alone (z = 2.29, *p* = 0.022) and TV width alone (z = 2.12, *p* = 0.034), while the difference between SNsum and TV width was not statistically significant (z = −0.59, *p* = 0.555). At the optimal cutoff value determined by maximizing Youden’s J index, the combined predictor yielded a sensitivity of 75.0% and specificity of 82.6%. At this cutoff the confusion matrix comprised 60 true positives, 20 false negatives, 4 false positives, and 19 true negatives, corresponding to a sensitivity of 75.0% (95% CI: 64.5–83.2%), specificity of 82.6% (95% CI: 62.8–93.0%), positive predictive value of 93.8%, negative predictive value of 48.7%, and overall accuracy of 76.7% (Figure 3C).

**Figure 4:**
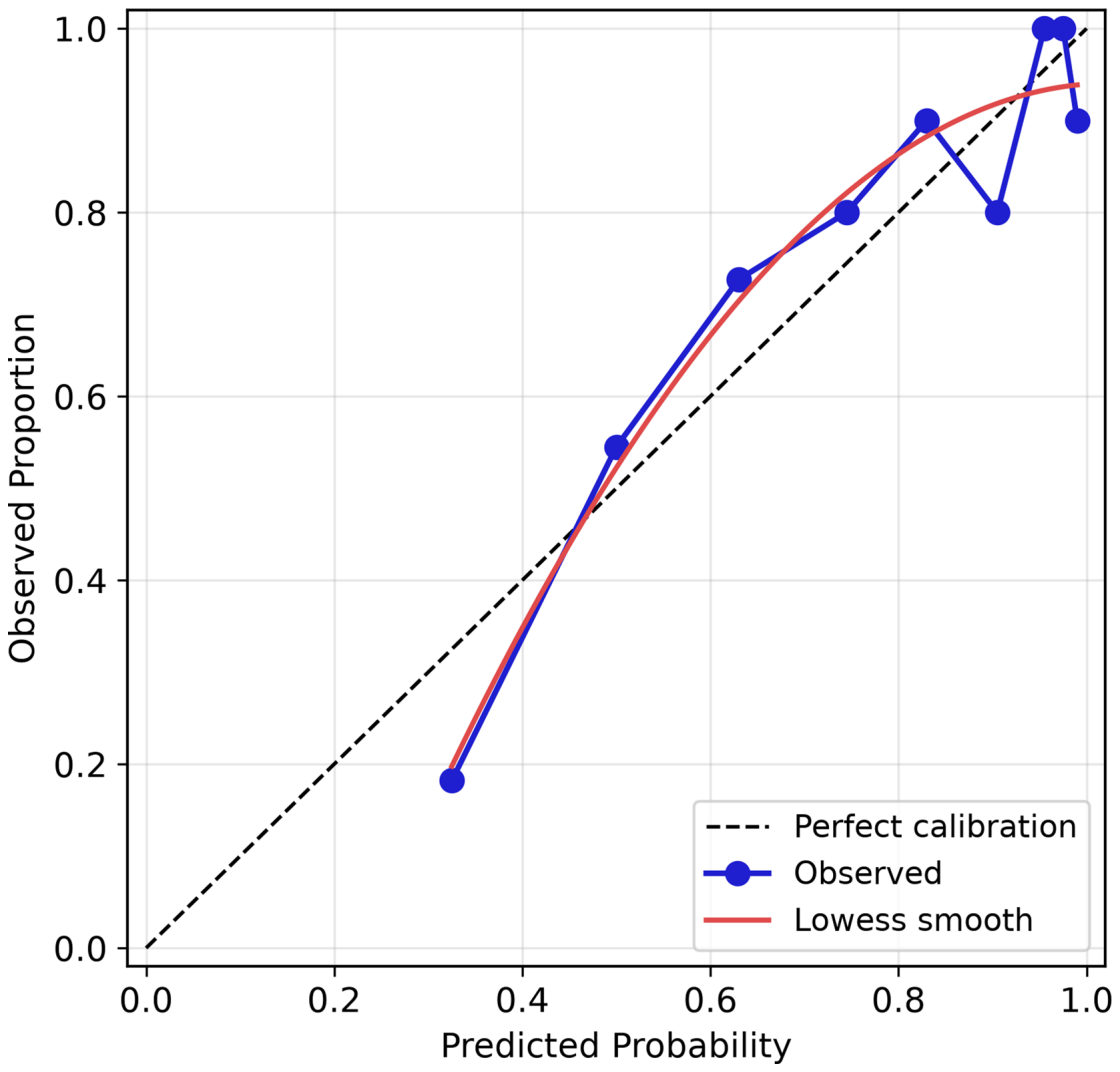
Calibration plot of the combined predictor model. Observed proportions of PD diagnosis are plotted against predicted probabilities across deciles. The dashed diagonal line represents perfect calibration. The proximity of observed values to the reference line, together with a non-significant Hosmer-Lemeshow test (χ^2^ = 11.046, *p* = 0.199), indicates acceptable calibration of the combined model.

Bootstrap internal validation with 1,000 resampling iterations yielded a mean optimism of 0.019, resulting in an optimism-corrected AUC of 0.830 (bootstrap 95% CI: 0.751–0.923), and the calibration plot demonstrated close agreement between predicted and observed probabilities across deciles (Figure 4). In the Firth penalized-likelihood sensitivity analysis, SNsum (OR = 18.72, 95% CI: 2.14–210.35, *p* = 0.009) and third ventricle width (OR = 2.31, 95% CI: 1.42–3.98, *p* < 0.001) remained independently associated with PD, with markedly narrower confidence intervals than the conventional model. Repeated stratified 10-fold cross-validation (five repeats) yielded a mean AUC of 0.812 (95% CI: 0.731–0.894) for the combined predictor. In the age-stratified analysis restricted to participants aged 65 years or older (66 PD and 15 ET), the combined predictor retained comparable discrimination (AUC = 0.837, 95% CI: 0.721–0.938). These sensitivity analyses are summarized in Table 4.

**Table 4:** Sensitivity analyses of the combined TCS predictor.

Analysis	Parameter/metric	Estimate	95% CI	*p*
Firth penalized logistic regression	SNsum (OR)	18.72	2.14–210.35	0.009
	TV width (OR)	2.31	1.42–3.98	< 0.001
	LN hyperecho. (OR)	2.98	0.51–17.40	0.223
	Age (OR)	0.96	0.89–1.03	0.284
Discrimination of combined predictor	Apparent AUC	0.848	0.743–0.932	—
	Bootstrap-corrected AUC	0.830	0.751–0.923	—
	Repeated 10-fold CV AUC	0.812	0.731–0.894	—
	Age-stratified (≥ 65 y) AUC	0.837	0.721–0.938	—
Confusion matrix at optimal cutoff	Sensitivity	75.0%	64.5–83.2%	—
	Specificity	82.6%	62.8–93.0%	—
	Positive predictive value	93.8%	85.0–97.5%	—
	Negative predictive value	48.7%	33.3–64.4%	—
	Accuracy	76.7%	67.3–84.5%	—

Firth penalized-likelihood logistic regression provides bias-reduced estimates under sparse-data conditions. The confusion matrix corresponds to 60 true positives, 20 false negatives, 4 false positives, and 19 true negatives at the Youden-optimal cutoff. AUC, area under the ROC curve; CV, cross-validation; OR, odds ratio; CI, confidence interval; SNsum, total substantia nigra hyperechogenicity area; TV, third ventricle; LN, lentiform nucleus.

## 4 Discussion

PD and ET represent the two most frequently encountered tremor syndromes in clinical practice. The current gold standard for diagnosing both conditions relies primarily on clinical symptoms and medical history (5, 6). However, during the early disease stages, the clinical manifestations of PD and ET often overlap substantially, rendering differential diagnosis particularly challenging and directly affecting subsequent treatment strategies and prognostic assessments. Misdiagnosis and missed diagnoses frequently arise from interpretive discrepancies regarding the nature of postural versus resting tremor and subtle motor impairment, ultimately increasing the burden on patients and healthcare systems (4). Moreover, the two conditions share demographic overlap in age distribution and symptom onset patterns, necessitating the incorporation of reliable biomarkers and neuroimaging tools to improve diagnostic precision (22). Among the available imaging modalities, DaT-SPECT and PET provide high diagnostic specificity but remain largely confined to tertiary medical centers owing to their technical complexity, extended examination duration, high costs, and patient immobilization requirements. In contrast, conventional cranial computed tomography and MRI are more readily accessible but demonstrate limited specificity for differentiating PD from ET in the absence of structural abnormalities (9). TCS represents a relatively novel neuroimaging technique that has undergone substantial evolution since its initial application in movement disorders, with advances in transducer technology and signal processing now permitting high-resolution imaging of deep cerebral structures through the temporal bone window (14–16).

The primary neuropathological hallmarks of PD involve degeneration of dopaminergic neurons in the nigrostriatal pathway and abnormal aggregation of alpha-synuclein deposits (23). SN hyperechogenicity on TCS constitutes the classic finding associated with PD (11) and has been validated across multiple studies as a biomarker of considerable value (12, 13). In the present study, TCS examinations were performed by sonographers with more than five years of specialized experience, employing state-of-the-art equipment and standardized protocols. Beyond qualitative assessment of SN echogenicity, we recorded quantitative parameters including bilateral SN hyperechogenicity areas, maximum hyperechogenicity area, total midbrain area, and the SN hyperechogenicity-to-midbrain area ratio. Our findings demonstrated that the SN hyperechogenicity total area (SNsum) in the PD group [median 0.30 (0.13–0.46) cm^2^] was significantly greater than in the ET group [median 0 (0–0.25) cm^2^], consistent with the findings of a recent systematic review and meta-analysis (12). Statistically significant differences between the two groups were also observed for bilateral SN hyperechogenicity areas, maximum SN hyperechogenicity area, and the SN-to-midbrain ratio after Bonferroni correction. No significant laterality differences were found for midbrain area between groups, suggesting that unilateral TCS examination may retain diagnostic value in patients with suboptimal temporal acoustic windows on one side. Notably, the ET group exhibited a non-zero upper quartile for SNsum (0.25 cm^2^), indicating that approximately 25% of ET patients also demonstrated measurable SN hyperechogenicity, which limits the specificity of SN evaluation as a standalone diagnostic criterion and underscores the necessity of a multi-parameter approach.

The principal contribution of this study is the integration of several TCS-accessible parameters, acquired within a single examination, into one age-adjusted combined predictor. Compared with prior TCS studies that relied predominantly on SN echogenicity alone (12, 17) and with longitudinal work reporting SN hyperechogenicity in both ET and PD (22), the present combined predictor added third ventricle width and formally quantified its incremental discrimination using pairwise DeLong testing, an approach seldom applied in previous PD-versus-ET TCS studies. This single-modality, multi-parameter strategy parallels the broader trend toward multimodal and machine-learning imaging frameworks in PD (18, 19), while retaining the accessibility and low cost of bedside ultrasound. These features, rather than any single measurement, constitute the intended novelty of the study.

In addition to SN evaluation, TCS enables assessment of thalamic-level LN echogenicity, TV width, and midbrain-level BR echogenicity. However, the diagnostic utility of these supplementary parameters in differentiating PD from ET has been addressed by relatively few systematic investigations, and the available evidence remains inconclusive. Prior research has suggested that BR echogenicity abnormalities detected by TCS correlate with depressive and anxiety symptoms in PD patients rather than with motor manifestations, while LN echogenicity intensity may provide diagnostic clues for symptomatic parkinsonism (24). In our study, the PD group demonstrated significantly greater TV width (7.31 ± 1.73 mm vs. 5.57 ± 1.47 mm) and higher prevalence of LN hyperechogenicity (33.8% vs. 8.7%) compared with the ET group, whereas no significant differences were observed in midbrain area or BR echogenicity between groups. The widening of the third ventricle in PD may reflect more extensive cerebral atrophy associated with the neurodegenerative process, which is consistent with MRI-based volumetric studies demonstrating progressive ventricular enlargement in PD relative to age-matched controls (16). However, since TV width also increases physiologically with age, we included age as a covariate in the multivariable logistic regression model. Age did not emerge as an independent predictor (OR = 0.946, *p* = 0.172), indicating that the association between TV width and PD persisted after accounting for age-related confounding. Nevertheless, ventricular enlargement is a nonspecific finding influenced by aging, cerebrovascular disease, and several neurodegenerative disorders; TV width should therefore be interpreted as a supportive rather than a disease-specific biomarker, informative only in combination with SN and clinical assessment.

Age-adjusted binary logistic regression analysis identified SNsum (OR = 69.023, 95% CI: 2.339–2037.215, *p* = 0.014) and TV width (OR = 2.531, 95% CI: 1.472–4.354, *p* = 0.001) as independent predictors of PD, while LN hyperechogenicity did not reach statistical significance after adjustment. Multicollinearity assessment using VIF analysis confirmed that SNsum and TV width were not collinear with each other (VIF = 1.175 and 1.255, respectively), supporting their simultaneous inclusion in the model. Based on these two independently significant parameters, a combined TCS predictor was constructed using the predicted probability from the logistic regression model. ROC curve analysis demonstrated that the combined predictor achieved an AUC of 0.848 (95% CI: 0.743–0.932). Importantly, pairwise DeLong tests confirmed that the combined predictor yielded a statistically significantly higher AUC than SNsum alone (z = 2.29, *p* = 0.022) or TV width alone (z = 2.12, *p* = 0.034), providing formal statistical evidence for the incremental diagnostic value of the multi-parameter approach beyond either parameter individually. At the optimal cutoff, the combined predictor yielded a sensitivity of 75.0% and specificity of 82.6%. While these performance metrics indicate moderate diagnostic capability, the sensitivity of 75.0% implies that approximately one-quarter of PD patients may be missed, and the specificity of 82.6% suggests that nearly one-fifth of ET patients could be misclassified. Therefore, the clinical utility of this combined predictor should be considered as an adjunctive tool complementing clinical assessment rather than a standalone diagnostic criterion.

To address potential overfitting given the limited sample size and low events-per-variable ratio (23 ET events / 5 variables = 4.6, below the recommended minimum of 10), we performed bootstrap internal validation with 1,000 resampling iterations. The resulting mean optimism of 0.019 and an optimism-corrected AUC of 0.830 suggest that the degree of overfitting is modest, though not negligible. Because the wide confidence interval for the SNsum odds ratio (2.339–2037.215) indicated unstable estimation under sparse-data conditions, we further applied Firth penalized-likelihood regression, which yielded bias-reduced and substantially narrower estimates for SNsum (OR = 18.72, 95% CI: 2.14–210.35) and TV width (OR = 2.31, 95% CI: 1.42–3.98) while preserving the direction and significance of both associations. Repeated 10-fold cross-validation produced a mean AUC of 0.812, concordant with the bootstrap-corrected estimate, indicating that the discrimination of the combined predictor was not an artifact of a single data partition. Additionally, the Hosmer-Lemeshow goodness-of-fit test yielded a non-significant result (χ^2^ = 11.046, *p* = 0.199), and the calibration plot (Figure 4) demonstrated reasonable agreement between predicted probabilities and observed outcomes across deciles, supporting the adequacy of model calibration. The notably wide confidence interval for the SNsum OR in the conventional model reflects the small sample size and sparse data in the ET group and should be interpreted with caution.

Several limitations of this study merit acknowledgment. First, this was a single-center retrospective observational study with a limited and unbalanced sample size (80 PD vs. 23 ET patients), which may introduce selection bias and limit the generalizability of the results. The low events-per-variable ratio in the logistic regression model, despite bootstrap and Firth penalized-likelihood correction, warrants cautious interpretation of the regression coefficients and their clinical application. Second, most PD patients had relatively established disease (median duration 39 months, with 60% at H-Y stage greater than 2), whereas the greatest diagnostic uncertainty arises in *de novo* or early-stage (H-Y stage 1–2) patients; the reported performance may therefore overestimate accuracy in early-stage cohorts, and validation in newly diagnosed populations is required. Third, approximately 8.3% of initially screened patients (10/120, predominantly elderly females) were excluded due to inadequate temporal bone windows with excessive acoustic attenuation, which represents an inherent limitation of TCS methodology and may introduce selection bias toward patients with better acoustic windows. Fourth, this study did not include a healthy control group, which would have been valuable for establishing normative reference ranges for TCS parameters. Fifth, TCS image quality and measurement accuracy are operator-dependent, and although our sonographers underwent standardized training, formal inter-observer and intra-observer reliability assessments (such as intraclass correlation coefficients) were not performed. Sixth, while SN hyperechogenicity demonstrates high sensitivity for PD, a proportion of ET patients also exhibited enhanced SN echogenicity (upper quartile of SNsum = 0.25 cm^2^ in the ET group), necessitating interpretation in conjunction with other clinical and imaging parameters. Above all, the model underwent only internal validation; it should be regarded as an exploratory predictor rather than a clinically validated diagnostic tool. These limitations underscore the need for future multicenter prospective studies with larger, balanced cohorts, healthy control groups, formal reliability assessments, and external validation of the combined predictor model. In conclusion, multi-regional multi-parameter TCS may serve as a practical and moderately sensitive neuroimaging tool for differentiating PD from ET; the combined predictor offered internally validated, statistically significant incremental discrimination that should be interpreted as exploratory and confirmed in larger, independent, multicenter cohorts with prospective external validation before it can be recommended for routine clinical use.

## Data Availability

The original contributions presented in the study are included in the article/supplementary material, further inquiries can be directed to the corresponding authors.

## Glossary

PD: Parkinson’s disease
ET: essential tremor
DaT-SPECT: dopamine transporter single photon emission computed tomography
PET: positron emission tomography
MRI: magnetic resonance imaging
TCS: transcranial sonography
SN: substantia nigra
BR: brainstem raphe
LN: lentiform nucleus
TV: third ventricle
ROC: receiver operating characteristic
UPDRS-III: the Unified Parkinson’s Disease Rating Scale Part III
H-Y: Hoehn-Yahr
SD: standard deviation
IQR: interquartile range
VIF: variance inflation factor
AUC: area under the ROC curve
OR: odds ratio
CI: confidence interval
PPV: positive predictive value
NPV: negative predictive value
